# Efficacy of personalized traditional Chinese medicine massage combined with tuina in the treatment of pediatric congenital heart disease and malnutrition intolerance

**DOI:** 10.12669/pjms.42.4.14839

**Published:** 2026-04

**Authors:** Yanhua Wang, Junping Yao, Yaqin Xiao, Qian Ma, Shuguang Tao

**Affiliations:** 1 Yanhua Wang, Department of Nursing Management, Hebei Children’s Hospital, The Fifth People’s Hospital of Hebei Province, Hebei Provincial Clinical Research Center for Pediatric Health and Diseases, Shijiazhuang, Hebei Province 050031, P.R. China; 2 Junping Yao Department of Cardiac Surgery, Department of Nursing Management, Hebei Children’s Hospital, The Fifth People’s Hospital of Hebei Province, Hebei Provincial Clinical Research Center for Pediatric Health and Diseases, Shijiazhuang, Hebei Province 050031, P.R. China; 3 Yaqin Xiao Department of Cardiac Surgery, Department of Nursing Management, Hebei Children’s Hospital, The Fifth People’s Hospital of Hebei Province, Hebei Provincial Clinical Research Center for Pediatric Health and Diseases, Shijiazhuang, Hebei Province 050031, P.R. China; 4 Qian Ma Department of Rehabilitation and Physical Therapy, Department of Nursing Management, Hebei Children’s Hospital, The Fifth People’s Hospital of Hebei Province, Hebei Provincial Clinical Research Center for Pediatric Health and Diseases, Shijiazhuang, Hebei Province 050031, P.R. China; 5 Shuguang Tao Department of Cardiac Surgery, Department of Nursing Management, Hebei Children’s Hospital, The Fifth People’s Hospital of Hebei Province, Hebei Provincial Clinical Research Center for Pediatric Health and Diseases, Shijiazhuang, Hebei Province 050031, P.R. China

**Keywords:** Chinese Medicine (TCM) massage, Congenital heart disease (CHD), Malnutrition intolerance, Personalized Traditional Tuina

## Abstract

**Objective:**

To evaluate personalized Traditional Chinese Medicine (TCM) massage combined with Tuina for children with congenital heart disease (CHD) and malnutrition intolerance.

**Methodology:**

Clinical data of 315 children from Hebei Children’s Hospital (December 2023 – May 2025) were retrospectively analyzed and divided into a study group (n=160, personalized TCM massage plus Tuina) and a control group (n=155, routine nursing). Symptoms, growth indicators, gastrointestinal hormones [motilin (MLT), gastrin (GAS), somatostatin (SS)], nutritional markers [hemoglobin (Hb), albumin (ALB)], and complications were compared.

**Results:**

The improvement rates of symptoms such as feeding difficulties, vomiting, diarrhea and abdominal distension in the research group were higher than those in the control group (P < 0.05). Post-intervention growth indicators and serum MLT, GAS, Hb, and ALB levels increased more significantly in the study group, while SS levels decreased further (P<0.05). The complication rate was lower in the study group (2.50%) than in the control group (8.39%) (P<0.05).

**Conclusion:**

Personalized TCM massage combined with Tuina effectively improves clinical symptoms, promotes growth, regulates gastrointestinal hormones, and enhances nutritional status in children with CHD and malnutrition intolerance, with fewer complications than routine nursing.

## INTRODUCTION

Congenital heart disease (CHD) affects 6%–10% of live births.^1^,^2^ Malnutrition intolerance, marked by feeding difficulties and growth retardation, is a common complication that impairs immunity and cardiac recovery while compromising prognosis.^3^-^5^ Conventional nutritional and pharmacological treatments often have limited efficacy and may cause adverse reactions, such as gut flora imbalance. 6,7

Traditional Chinese medicine (TCM) identifies this condition as “heart-spleen deficiency” or “qi-blood insufficiency,” stemming from dysfunction in the spleen-stomach transport.^8^ As non-invasive “green therapies,” TCM massage and Tuina facilitate meridian regulation and qi replenishment.^9^ However, clinical evidence for their combined application in CHD-related malnutrition remains scarce.

This study aimed to assess the effectiveness of personalized TCM massage plus Tuina in children with CHD and malnutrition intolerance.

## METHODOLOGY

Clinical data of 315 children with CHD and malnutrition intolerance from Hebei Children’s Hospital (December 2023 – May 2025) were retrospectively analyzed. Patients were assigned to either a study group (n=160, personalized TCM massage plus Tuina) or a control group (n=155, routine nursing).

### Ethical approval:

The ethics committee of our hospital approved this study with number 2024-0721; Date: March 6, 2024.

### Inclusion Criteria:

- Aged 1-19 months.
- Confirmed CHD (VSD, ASD, PDA, or TOF) via echocardiography.^10^
- Manifestations of malnutrition intolerance (feeding difficulty, vomiting, diarrhea, abdominal distension, or growth retardation) for ≥1 month.
- Stable cardiac function (NYHA I–III) without acute heart failure.
- Complete clinical data.

### Exclusion Criteria:

- Severe hepatic, renal, immune, or coagulation disorders.
- Non-cardiac malabsorption, including gastrointestinal malformations or metabolic diseases.
- Local skin lesions, infections, or allergic constitution.
- Severe complications such as hypoxia, pulmonary hypertension, or endocarditis.

### Routine Nursing Intervention:

Standardized care for the control group included personalized nutrition (small, frequent meals with breast milk or high-calorie formula), monitoring of vital signs and growth, probiotics, and health education. Postural guidance or nasogastric feeding was provided for children with feeding difficulties.

### Personalized Traditional Chinese Medicine (TCM) Massage Combined with Tuina Intervention:

Daily 15–20-minute sessions were performed by certified nurses (>5 years’ experience) following standardized training and specialist supervision, with >95% compliance. Based on syndrome differentiation, personalized massage targeted specific acupoints (3–5 min/point):

- Heart-Spleen Deficiency (feeding difficulty, loose stools): kneading ST36, CV12, BL20, and BL15;
- Spleen-Stomach Disharmony (vomiting, distension): pushing PC6, LI4, ST36, and CV13;

### Qi-Blood Insufficiency (growth retardation, pale nails):

tonifying SP10, SP6, CV6, and CV4. Supplementary Tuina included clockwise abdominal massage (5–8 min), back kneading along the Bladder Meridian (5–10 repeats) with spine pinching (3–5 times), and extremity kneading (3–5 min). Safety protocols mandated using moderate force, avoiding cardiac areas, and suspending sessions for distress such as crying, cyanosis, or tachycardia; post-intervention rest (10–15 min) and delayed feeding were strictly enforced.

### Observation Indicators:

Symptom severity was graded using a structured system:

- Feeding difficulty (Severe: ≥30 min, refusal ≥3/day; Moderate: 20–30 min, 1–2/day; Mild: ≤20 min, occasional);
- Vomiting (Severe: ≥3/day; Moderate: 1–2/day; Mild: 1–3/week);
- Diarrhea (Severe: ≥7/day; Moderate: 5–6/day; Mild: 3–4/day);

Abdominal distension (Severe: persistent/tympanic; Moderate: 1–2/day; Mild: occasional). Inter-rater reliability was ensured via standardized training and dual-log verification. The improvement rate was calculated based on the reduction of severe and moderate cases. Growth indicators (weight, length, head circumference, weight gain rate) were recorded at four weeks, with normal development defined as falling within the 3rd–97th percentiles on growth curves. Additionally, serum gastrointestinal hormones (MLT, GAS, SS), nutritional markers (Hb, ALB), and complication incidence (aspiration pneumonia, diarrhea, skin allergies) were assessed pre- and post-intervention.

### Statistical analysis:

Data were analyzed using SPSS 25.0. The sample size (n=315) was determined by all consecutive cases meeting the inclusion criteria during the study period. A post-hoc power analysis based on feeding difficulty improvement (87.50% vs. 76.77%) indicated that with α = 0.05 the target power (0.80) required a minimum of 138 cases per group. The actual sample sizes in the study (n=160 and n=155) provided a statistical power exceeding 0.80. Measurement data (*x̅*+*s*) were compared via t-test, while categorical data [n(%)] were analyzed using the test. P<0.05 was considered statistically significant.

## RESULTS

Baseline characteristics, including demographics, CHD type, and clinical manifestations, showed no significant differences between groups (P > 0.05, Table-I). The improvement rates of symptoms such as feeding difficulties, vomiting, diarrhea and abdominal distension in the research group were higher than those in the control group (P < 0.05, Table-II). Post-intervention increases in weight, length, and head circumference were significantly greater in the study group (P < 0.05, Table-III).

**Table-I:** Comparison of General Data Between the Two Groups.

Item	Study Group (n=160)	Control Group (n=155)	t/χ^2^	P
***Gender[n(%)]***				
Male	86(53.75)	80(51.61)	0.144	0.704
Female	74(46.25)	75(48.39)		
Age(*x̅*+*s*, months)	9.25±3.68	9.56±3.45	0.771	0.441
Body Weight(*x̅*+*s*, kg)	5.32±1.56	5.15±1.48	0.992	0.322
Gestational Age (*x̅*+*s*, weeks)	38.51±1.24	38.29±1.30	1.537	0.125
Birth Weight(*x̅*+*s*, kg)	2.93±0.48	2.86±0.51	1.255	0.211
***CHD Type[n(%)]***				
Ventricular Septal Defect	62(38.75)	59(38.06)	0.370	0.946
Atrial Septal Defect	48(30.00)	45(29.03)		
Patent Ductus Arteriosus	30(18.75)	28(18.06)		
Tetralogy of Fallot	20(12.50)	23(14.84)		
***NYHA Classification [n(%)]***						
Grade-I	45(28.13)	42(27.10)	0.095	0.954
Grade-II	82(51.25)	79(50.97)		
Grade-III	33(20.63)	34(21.94)		
Course of Malnutrition Intolerance(*x̅*+*s*, months)	3.22±1.15	3.08±1.09	1.108	0.269
***Manifestations of Malnutrition Intolerance[n(%)]***				
Feeding Difficulty	55(34.38)	50(32.26)	0.159	0.690
Vomiting	51(31.88)	46(29.68)	0.178	0.673
Diarrhea	48(30.00)	50(32.26)	0.187	0.665
Abdominal Distension	46(28.75)	49(31.61)	0.306	0.580

**Table-II:** Comparison of Improvement of Malnutrition Intolerance Symptoms Between the Two Groups [n(%)].

Group	Improvement Rate of Feeding Difficulty	Improvement Rate of Vomiting	Improvement Rate of Diarrhea	Improvement Rate of Abdominal Distension
Study Group	92.73(51/55)	92.16(47/51)	93.75(45/48)	93.48(43/46)
Control Group	78.00(39/50)	76.09(35/46)	80.00(40/50)	79.59(39/49)
*χ^2^*	4.639	4.778	4.024	5.020
*P*	0.031	0.029	0.045	0.025

**Table-III:** Comparison of Growth and Development Indicators Between the Two Groups(*x̅*+*s*).

Time	Group	Number of Cases	Body Weight (kg)	Body Length (cm)	Head Circumference (cm)	Weight Gain Rate (g/week)
Before Intervention	Study Group	160	7.32±1.56	65.23±4.15	42.11±2.36	/
	Control Group	155	7.15±1.48	64.87±4.02	41.89±2.28	/
	*t*		0.992	0.782	0.841	/
	*P*		0.322	0.435	0.401	/
After Intervention	Study Group	160	8.95±1.62	69.85±4.26	44.32±2.41	40.75±8.23
	Control Group	155	7.86±1.53	67.32±4.11	43.15±2.35	32.52±7.68
	*t*		6.135	5.362	4.361	9.169
	*P*		0.000	0.000	0.000	0.000

The study group exhibited significantly better biochemical profiles post-intervention, with higher MLT, GAS, Hb, and ALB levels alongside lower SS levels (P < 0.05, Table-IV). The complication rate in the study group was significantly lower than in the control group (2.50% vs. 8.39%; P < 0.05, Table-V).

**Table-IV:** Comparison of Gastrointestinal Function andNutrition-Related Indicators Between the Two Groups(*x̅*+*s*).

Time	Group	Number of Cases	MLT(pg/ml)	GAS(pg/ml)	SS(pg/ml)	Hb(g/L)	ALB(g/L)
Before Intervention	Study Group	160	185.62±32.45	86.25±15.32	45.68±12.35	105.62±12.38	32.56±3.46
	Control Group	155	182.35±30.68	84.76±14.89	44.23±11.86	103.45±11.89	31.89±3.28
	*t*		0.918	0.875	1.005	1.586	1.763
	*P*		0.359	0.382	0.316	0.114	0.079
After Intervention	Study Group	160	235.87±35.62	112.35±16.48	18.75±8.62	118.39±13.48	36.89±3.62
	Control Group	155	219.56±29.45	104.62±15.76	25.32±9.45	108.62±12.76	33.45±3.51
	*t*		4.422	4.252	6.497	6.602	8.559
	*P*		0.000	0.000	0.000	0.000	0.000

**Table-V:** Comparison of Complications Between the Two Groups[n(%)].

Group	Number of Cases	Aspiration Pneumonia	Aggravated Diarrhea	Skin Allergy	Total Incidence
Study Group	160	2(1.25)	1(0.63)	1(0.63)	4(2.50)
Control Group	155	5(3.23)	6(3.87)	2(1.29)	13(8.39)
*χ^2^*					5.344
*P*					0.021

## DISCUSSION

This study confirms that personalized TCM massage, combined with Tuina, significantly improves clinical symptoms, promotes growth, and enhances nutritional status in children with CHD and malnutrition intolerance compared with routine nursing care. Notably, this integrated approach significantly regulates gastrointestinal hormone secretion and is associated with a lower incidence of complications.

Children with CHD are susceptible to malnutrition intolerance due to cardiac insufficiency-driven gastrointestinal congestion and impaired digestive enzyme secretion.^11^,^12^ Although routine nursing—focusing on nutritional support and feeding adjustments—can partially alleviate symptoms, it often fails to fundamentally resolve underlying malabsorption.^13^

Our results show symptom improvement rates reached 92.16%–93.48% in the study group (P<0.05), aligning with previous findings on TCM external therapies.^14^,^15^ Rooted in the TCM concept of “infantile malnutrition” caused by spleen-stomach weakness, stimulation of key acupoints—Zusanli (ST36), Zhongwan (CV12), and Pishu (BL20)—revitalizes transport and transformation functions.^8^ This likely facilitates neurohumoral regulation, augmenting peristalsis and enzyme secretion while relieving smooth muscle spasms.^16^

Significant gains in weight, length, and head circumference (P<0.05) support the TCM principle that the “spleen is the foundation of acquired constitution”.^17^-^20^ Improved spleen-stomach function ensures adequate Qi and blood production for physical development and subsequent surgical tolerance.^21^,^22^ Furthermore, the strong correlation between quantitative symptom scores and objective biochemical markers (MLT, GAS) validates the clinical utility and construct validity of our criteria.

Gastrointestinal hormones (MLT, GAS, SS) are pivotal in digestive regulation.^23^-^25^ In this study, increased MLT and GAS alongside decreased SS levels (P<0.05) suggest that the intervention effectively restores hormonal balance.^26^,^27^ This neurohumoral stability, likely mediated via vagal nerve reflexes, depends on high intervention adherence.^28^ Continuous daily stimulation prevents hormonal fluctuations, while elevated Hb and ALB levels confirm enhanced iron absorption and protein synthesis.

The significantly lower complication rate in the study group (2.50% vs. 8.39%) underscores the safety of this non-invasive approach.^28^ Enhanced gastrointestinal motility and improved nutritional status likely reduced risks of indigestion, aspiration, and secondary infections (such as pulmonary or incision infections). This validates the intervention as a safe alternative to conventional pharmacological treatments.

### Limitations:

This is a retrospective, single-center study with a small sample size, which increases the risks of selection bias and unmeasured confounders, such as home caregiving and dietary compliance. Despite strict exclusion criteria, unidentified factors may still influence outcomes. The 4-week observation period did not allow for the evaluation of long-term durability or recurrence. Nutritional assessment relied solely on hemoglobin and albumin, omitting micronutrients such as vitamin D, zinc, and calcium. Furthermore, the retrospective nature of the study prevented a formal assessment of parent-reported outcomes, including quality of life. Potential bias from emotional responses, the absence of a sham/placebo control, and the lack of standardized cooperation scores limit behavioral analysis, although objective biochemical improvements support biological efficacy. Finally, small sample sizes of complex CHD types, such as Tetralogy of Fallot, precluded formal anatomical subgroup analysis. Univariate analysis was prioritized over multivariate regression to avoid model overfitting and maintain statistical power.

## CONCLUSION

Personalized TCM massage combined with Tuina can effectively alleviate clinical symptoms in children with CHD and malnutrition intolerance, promote growth and development, regulate gastrointestinal hormone secretion, enhance nutritional status, and reduce the incidence of complications. It is a safe and effective nursing intervention that needs to be promoted.

### Recommendations:

Future research should prioritize multi-center prospective RCTs with longitudinal follow-up to validate clinical durability. Comprehensive assessments should incorporate micronutrient markers (vitamin D, iron, zinc) and the Pediatric Quality of Life Inventory (PedsQL) to evaluate quality of life. Larger cohorts are needed to analyze CHD anatomical subtypes. Molecular studies should explore hormone-regulating signaling pathways. Finally, investigating synergistic effects with probiotics and enteral nutrition will help optimize nursing protocols.

### Authors’ contributions:

**YW and JY:** Literature search, study design and manuscript writing.

**YX, QM and ST:** Data collection, data analysis and interpretation. Critical Review

**YW and JY:** Manuscript revision and validation and is responsible for the integrity of the study.

All authors have read and approved the final manuscript.
